# SG-APSIC1069: Implementing infection prevention bundle significantly reduced multidrug-resistant organisms infection and healthcare-associated infections in intensive care unit at a national hospital in Vietnam

**DOI:** 10.1017/ash.2023.74

**Published:** 2023-03-16

**Authors:** Thang Phung, Thoa Vo Thi Hong, Ven Le Thi, Dung Phan Tien, Hung Le Quoc

**Affiliations:** Cho Ray Hospital, Ho Chí Minh City, Vietnam; Cho Ray Hospital, Ho Chí Minh City, Vietnam; Cho Ray Hospital, Ho Chí Minh City, Vietnam; Cho Ray Hospital, Ho Chi Minh City, Vietnam; Cho Ray Hospital, Ho Chi Minh City, Vietnam

## Abstract

**Objectives:** In Vietnam, the burden of healthcare-associated infections (HAIs), especially by multidrug-resistant organisms (MDROs), can be greater at national hospitals where a high number of severe patients from lower-tier hospitals are received for treatment. To reduce MDRO and HAI incidences at the tropical diseases intensive care unit (ICU) of Cho Ray Hospital, the final line of treatment for 20 southern provinces of Vietnam, a comprehensive infection prevention bundle was developed and implemented in 2019. In this retrospective study, we evaluated the effectiveness of this intervention in preventing MDRO infections and HAIs among patients at risk. **Methods:** The infection prevention bundle included elements to improve administration controls, environmental controls, and personal protective equipment usage. The bundle was implemented via training and active monitoring. Medical data, such as microbiology results, length of hospital stay, treatment cost of all patients admitted to the targeted ICU, and data on adherence to the bundle elements, were collected via routine monitoring from July to December 2019. These data were reviewed and analyzed. An independent 2-sample *t* test was used to calculate the significance of the differences in MDRO and HAI rates before and after the intervention. **Results:** The mean number of MDRO infections decreased significantly after implementation of the infection prevention bundle (7.0 vs 3.3; *P* = .011). HAI and ventilator-associated pneumonia (VAP) rates also decreased significantly (5.9 vs 3.7; *P*= .013 and 20.4 vs 13.7; *P*=0.047, respectively). The mean total treatment cost per patient was reduced by 1.8 million VND (US $76.76). Bundle-element adherence was high throughout the intervention period, ranging from 72.7% (putting MDRO sign on beds) to 100% (hand hygiene, cohort patients, environment cleaning). **Conclusions:** Implementation of an appropriate infection prevention bundle with a high adherence rate by healthcare workers helped to effectively reduce MDRO infection and HAI rates in the tropical diseases ICU.

